# Rice-Husk-Ash-Based Geopolymer Coating: Fire-Retardant, Optimize Composition, Microstructural, Thermal and Element Characteristics Analysis

**DOI:** 10.3390/polym13213747

**Published:** 2021-10-29

**Authors:** Mohd Salahuddin Mohd Basri, Faizal Mustapha, Norkhairunnisa Mazlan, Mohd Ridzwan Ishak

**Affiliations:** 1 Department of Process and Food Engineering, Faculty of Engineering, University Putra Malaysia (UPM), Serdang 43400, Selangor, Malaysia; 2 Laboratory of Halal Science Research, Halal Products Research Institute, Universiti Putra Malaysia (UPM), Serdang 43400, Selangor, Malaysia; 3 Laboratory of Biopolymer and Derivatives, Institute of Tropical Forestry and Forest Products (INTROP), Universiti Putra Malaysia (UPM), Serdang 43400, Selangor, Malaysia; 4 Department of Aerospace Engineering, Faculty of Engineering, University Putra Malaysia (UPM), Serdang 43400, Selangor, Malaysia; faizalms@upm.edu.my (F.M.); norkhairunnisa@upm.edu.my (N.M.); mohdridzwan@upm.edu.my (M.R.I.); 5 Institute of Advanced Technology (ITMA), Institute of Advanced Technology, Universiti Putra Malaysia, Serdang 43400, Selangor, Malaysia

**Keywords:** rice husk ash, geopolymer, coating, thermal properties, fire retardant, optimum factor, response surface methodology (RSM)

## Abstract

Geopolymer using aluminosilicate sources, such as fly ash, metakaolin and blast furnace slag, possessed excellent fire-retardant properties. However, research on the fire-retardant properties and thermal properties of geopolymer coating using rice husk ash (RHA) is rather limited. Additionally, the approach adopted in past studies on geopolymer coating was the less efficient one-factor-at-a-time (OFAT). A better approach is to employ statistical analysis and a regression coefficient model (mathematical model) in understanding the optimum value and significant effect of factors on fire-retardant and thermal properties of the geopolymer coating. This study aims to elucidate the significance of rice husk ash/activated alkaline solution (RHA/AA) ratio and NaOH concentration on the fire-retardant and thermal properties of RHA-based geopolymer coating, determine the optimum composition and examine the microstructure and element characteristics of the RHA-based geopolymer coating. The factors chosen for this study were the RHA/AA ratio and the NaOH concentration. Rice husk was burnt at a temperature of approximately 600 °C for 24 h to produce RHA. The response surface methodology (RSM) was used to design the experiments and conduct the analyses. Fire-retardant tests and thermal and element characteristics analysis (TGA, XRD, DSC and CTE) were conducted. The microstructure of the geopolymer samples was investigated by using a scanning electron microscope (SEM). The results showed that the RHA/AA ratio had the strongest effect on the temperature at equilibrium (TAE) and time taken to reach 300 °C (TT300). For the optimization process using RSM, the optimum value for TAE and TT300 could be attained when the RHA/AA ratio and NaOH concentration were 0.30 and 6 M, respectively. SEM micrographs of good fire-resistance properties showed a glassy appearance, and the surface coating changed into a dense geopolymer gel covered with thin needles when fired. It showed high insulating capacity and low thermal expansion; it had minimal mismatch with the substrate, and the coating had no evidence of crack formation and had a low dehydration rate. Using RHA as an aluminosilicate source has proven to be a promising alternative. Using it as coating materials can potentially improve fire safety in the construction of residential and commercial buildings.

## 1. Introduction

Steel is used in many areas as a major material in building construction, offshore structure, ships, bridges and airports. Despite its exceptional strength, steel is normally not able to withstand high temperatures between 470 and 500 °C, causing it to lose its strength slowly [1]. In building structures, failure of the load-bearing members of steel structures will lead to parts’ disintegration. Due to the presence of hydrocarbon and natural gas, a gas-pipe explosion in a building can occur if the pipe ruptures. For safety reasons, fire-retardant materials are essentially used as shields or coating to maintain the temperature of steel structures below 500 °C, in case of fire incidents [2].

There are two commonly used materials in steel coatings, namely cementitious materials and intumescent coating. The former is usually inorganic, not combustible when in contact with fire, durable and inexpensive. However, the coating from these materials has to be thick and thus too heavy to provide sufficient protection on the substrate. Intumescent coating, such as sodium silicate–based paint, is normally light, aesthetic and smooth and can be applied thinly. Its only drawbacks are poor water resistance and the tendency to form its original constituents, which are sodium carbonate and silicon dioxide, when reacting with carbon dioxide [3].

The intumescent coating expands when exposed to heat and creates a char layer to protect the steel substrate. Intumescence occurs from the interaction of three components: (i) an inorganic acid, such as ammonium polyphosphate (APP); (ii) a carbonaceous char-forming material, such as polyols; and (iii) a blowing agent, such as melamine [4]. Appropriate composition in making intumescent coating is crucial in producing the best performance for the intumescent system. Geopolymer has the potential as an intumescent coating [5,6,7,8].

To make the composites more environmentally friendly, the use of SiO_2_-rich materials as alternative is necessary. Abundant agricultural wastes containing high content of SiO_2_, such as rice husk ash (RHA), are viewed as cleaner and greener substitutes for improving the performance of geopolymeric composites. Most of the world’s rice is produced in Asia, and Asians account for more than 80 percent of global rice consumption. Rice production is estimated to be 156 million tons per year, resulting in the rice industry generating massive amount of solid waste [9]. RHA is a by-product obtained by burning rice husk for several heating purposes. RHA burned at 600 °C for 2 h, yielded a light grey husk [10]. Burning temperatures below 700 °C can be used to produce amorphous silica, which has a large surface area. When burning with oxygen at temperatures lower than 500 °C for an extended period, amorphous silica can be produced as well [11]. It comprises mostly the reactive amorphous SiO_2_ phase, depending on the burning process [12]. It is available and suitable for silicate polymeric condensation [13]. In building sector, geopolymer concrete led to reduced carbon dioxide emission compared to the ordinary Portland cement (OPC) mortar. Thus, it helps decrease the pollution problems created through its disposal in landfills, especially in important rice-producing countries such as in Thailand and India.

Geopolymer is an amorphous solid inorganic polymer with a tri-dimensional aluminosilicate structure produced through geopolymerization, the chemical reaction between alkaline solution and aluminosilicate source [14]. Biomass wastes, such as rice husk ash and palm oil fuel ash, have been recycled as energy sources [6,15]. Other common aluminosilicate sources include metakaolin, fly ash and ground granulated blast-furnace slag (GBBS) [16,17,18]. Geopolymer using an aluminosilicate source has shown excellent fire-retardant properties. In 1970, Davidovits [19] developed a geopolymer material as a fire-resistant system in buildings following a tragic fire which occurred in Europe. Later in 2008, a study was conducted on the fire performance of three different types of geopolymer, namely Na-poly(sialate), K-poly(sialate-siloxo) and K-poly(sialate-disiloxo) with a Si/Al ratio of 1, 2 and 3 respectively [20]. The fire-resistant test was conducted by using a direct flame at 1000 °C, on a 10 mm thick geopolymer composite panel. The non-exposed surface temperature was subsequently measured after 30 min. Exposed surface refers to the surface that is directly in contact with the flame. The surface at the back of the geopolymer composite panel is referred to as the non-exposed surface.

Rickard et al. [21] investigated the thermal expansion and thermal stability of five different types of fly ash (FA) in order to find out the potential of the geopolymer paste activated by sodium aluminate as a thermal barrier material. The composition of the amorphous component of each fly ash affected by the thermal test was determined by conducting XRF and XRF tests. The combined results showed that the thermal performance of geopolymer was strongly influenced by the Si/Al ratio. Si/Al ratio of higher than five produced geopolymers with excellent dimensional stability during heating at 1000 °C, while those with ratios lower than two (2) showed poor thermal stability. At high temperatures between 500 and 850 °C, sharp shrinkage occurred in all samples due to the compaction and densification of the samples by heat and the continuous steady motion of the aluminosilicate particles.

For lightweight panel applications, Abdul Rashid et al. [22] investigated the fire resistance of composite coatings reinforced with biofillers derived from geopolymer materials. The coating formulation was optimized in terms of thickness, alkaline activator ratio and curing regime, resulting in a RHA-based geopolymer that remained intact for nearly two hours when exposed to flame for a duration of one hour and forty minutes. They concluded that a geopolymer-binder-type composite is a plausible fire-resistant coating for structural insulated panels.

Sarazin et al. [7] investigated the fire resistance of novel geopolymer (GP) foams based on alkali-activated metakaolin and silica fume (SF). Fresh GP foams were applied as coatings on steel plates. After one week of curing, the foams were subjected to a flame burn-through test. Results showed that GP foams are excellent thermal barriers which recorded a temperature difference of 251 °C when compared to the uncoated steel plates. Le and Louda [23] studied the effect of time and temperature on the strengths and fire resistance of geopolymer foam (GF) coated on an aluminum plate. The composition of the GF used in the experiment included a potassium activator, basalt ground fiber and aluminum powder with a mass ratio to the binder of 0.45, 0.3 and 0.15, respectively. The results showed that the GF exhibited a substantial increase after three days in its compressive strength by 122.9% at the optimal temperature of 85 °C for two hours.

Geopolymer has shown to have excellent fire-retardant properties in studies using aluminosilicate sources. However, research on its fire-retardant properties and thermal properties of geopolymer coating using RHA and statistical analysis tools is rather limited. Studies conducted to identify the impact of several factors on the properties of the RHA-based geopolymer coating is also still minimal. In addition, recent studies have not adopted the improved method in determining the optimum formulation through statistical optimization.

Despite many publications on rice-husk-ash-based polymer composite reported in the literature in recent years [5,8,24,25,26], most studies were carried out on the one-factor-a-time (OFAT) approach. The application of statistical analysis and regression coefficients (mathematical models) is required effectively identify the optimal composition of geopolymer coating with improved fire-retardant and thermal properties to increase efficiency. The design of experiment (DOE) offers numerous advantages over the OFAT approach, including low resource requirements (experimental runs, time, materials and human resources), accurate measurement of main effects and interactions and the ability to simultaneously analyze multiple variables [27]. Furthermore, the RSM, initially coined by Box and Wilson [28], is widely used as a mathematical model for investigating significant effects, interactions and optimization conditions. The central composite design (CCD) is the most effective model for analysis and design [29]. Table 1 shows the difference in the total number of experimental runs between the full design of RSM and the full factorial (classical method) design based on 5-level factors. The former requires only 31 experimental runs (with one replication) for analyzing four factors, whereas a full factorial design requires 625 experimental runs to arrive at a comparable result [24].

Since the RSM approach, specifically the CDD, has been widely used in polymer optimization [30,31,32,33], it was thus adopted in this study. The objectives of this paper are to identify the significant effect of different factors (RHA/AA ratio and NaOH concentration) on the fire-retardant and thermal properties of RHA-based geopolymer coating, to determine the optimum composition for the RHA-based geopolymer coating and to study the microstructure and element characteristics of the RHA-based geopolymer coating.

## 2. Materials and Methods

### 2.1. Factors and Levels of the Design of Experiment (DOE)

In the study, the ratio of rice husk ash/activated alkaline solution (RHA/AA) and the sodium hydroxide (NaOH) concentration, designated as V_1_ and V_2,_ respectively, were chosen as factors. Based on the preliminary results using a screening process with fractional factorial design (FrFD), other factors, such as the ratio of AA solution, curing temperature and curing time, were kept constant at 5.5, 70 °C and seven days, respectively. Factors and levels used in the DOE are shown in Table 2.

### 2.2. Design of Experiment

At each design stage, five levels and two factors were applied in the CCD and with two replications for a total of 26 experimental runs. The factors were selected based on preliminary lab work, their significant effect on the responses and their working range (workability). Table 3 displays the complete CCD with coded and uncoded levels of these factors. The value for the total block is 1, with the experiments carried out in randomized order.

The optimization of RHA-based geopolymer coating was conducted by using Minitab @ 16.2 (Minitab, LLC, State College, PA, USA). An analysis of variance (ANOVA) was used to calculate the significance of the main factors and their interactions. The value of 95% was set as the significance level, which reflected the *p*-value of 0.05. Based on the correlation coefficient (R^2^) value, the regression coefficient model (mathematical model) developed in the ANOVA table was used for optimization. To acquire the regression coefficient model, experimental data were fitted with the second-order polynomial model. The general mathematical model obtained from the analysis is shown in Equation (1),

(1) $$\Upsilon =\beta_{0}+\sum_{t=1}^{3}\beta_{i}{Χ}_{i}+\sum_{i}^{3}\beta_{ii}{Χ}_{i}^{2}+\sum_{i-1}^{2}\sum_{j=i+1}^{3}\beta_{ij}{Χ}_{i}{Χ}_{j}$$

where Υ is the response; β_0_, β_i_, β_ii_ and β_ij_ are regression coefficients for the intercept, linear, quadratic and interaction terms, respectively; and X_i_ and X_j_ are coded values for the independent variables [34].

### 2.3. Raw Materials and Sample Preparation

**Figure 1:**
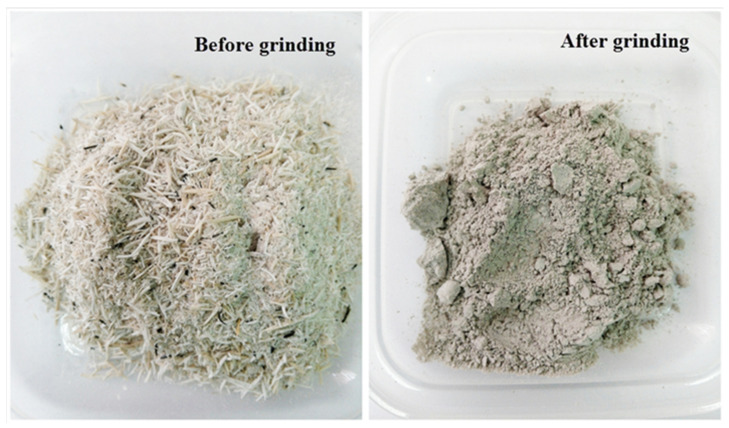
Images of RHA before (**left**) and after (**right**) grinding.

RHA was obtained from Maero Tech Sdn. Bhd (Nilai, Malaysia). Rice husk was burnt at a temperature of approximately 600 °C for 24 h to produce RHA. The material was ground, using a planetary mill (Pulverisette 4, FRITSCH GmbH—Milling and Sizing, Idar-Oberstein, Germany), and sieved through 75 micron opening to obtain finer particle sizes. Figure 1 shows images of RHA before and after grinding.

**Figure 2:**
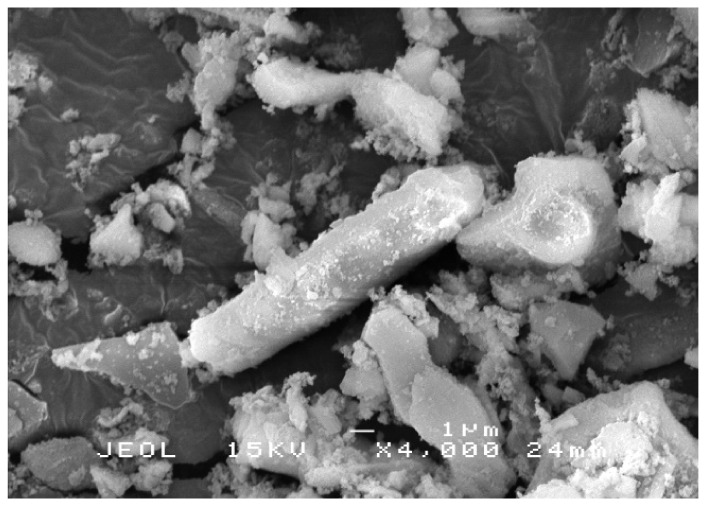
SEM image of RHA structure after grinding.

The fine structure of RHA before and after grinding was viewed under a S-3400N scanning electron microscope (SEM) (Hitachi, Tokyo, Japan). RHA particle size before grinding ranges from 1 to 100 μm. Particles appear as plates and thin shell-like structures with rectangular indents on the surface. These forms constitute the initial structure of the RH. RHA has porous, cellular surfaces due to its sponge-like character. Figure 2 shows the RHA structure after grinding. The particles contain high silica content with amorphous shapes similar to cristobalite and trace crystalline quartz [35].

In order to develop pozzolanic activity, RHA was ground to a very fine particle size [36]. The condition for burning RH is vital in producing the highest silica RHA in an amorphous state. Conversely, silica derived from unchecked incineration (temperatures higher than 700 to 800 °C) comprises mainly cristobalite and tridymite, which are non-reactive silica minerals [37]. The physical properties of the RHA after grinding are given in Table 4. The color of RHA was determined visually, and a hexadecimal (HEX) color code was generated by using a vector graphics editor and design program (Adobe Illustrator, Adobe, Inc., San Jose, CA, USA). A HEX color code of #d3d3d3 was determined, indicating that the color is light gray.

Sodium hydroxide (NaOH) and sodium silicate (Na_2_SiO_3_) were purchased from Evergreen Engineering & Resources (Semenyih, Malaysia). The sodium-based solution was chosen over potassium-based, due to lower material cost [38] and better mechanical properties as reported in previous studies [39]. The sodium silicate solution was purchased from LGC Scientific Sdn Bhd (Selangor, Malaysia). The chemical composition (by wt.%) of the solution was Na_2_O = 11.9%, SiO_2_ = 57.8% and H_2_O = 30.3%. Sodium hydroxide pellets with 97% purity were provided by Merck KGaA (Darmstadt, Germany). Different concentrations expressed as molarity, M of NaOH solution were prepared based on the amount of pellets dissolved in de-ionized water.

Mild steel plates with a thickness of 1 ± 0.05 mm and dimensions of 100 mm in length and 100 mm in width were cleaned by using sandpaper to improve the surface roughness and then washed with acetone to remove any unwanted oils or greases. Once the surface was dried at room temperature, the plates were placed in an oven at 45 °C for further drying to remove excess water. These were then used as substrate and coated with rice-husk-ash-based geopolymer coating.

**Figure 3:**
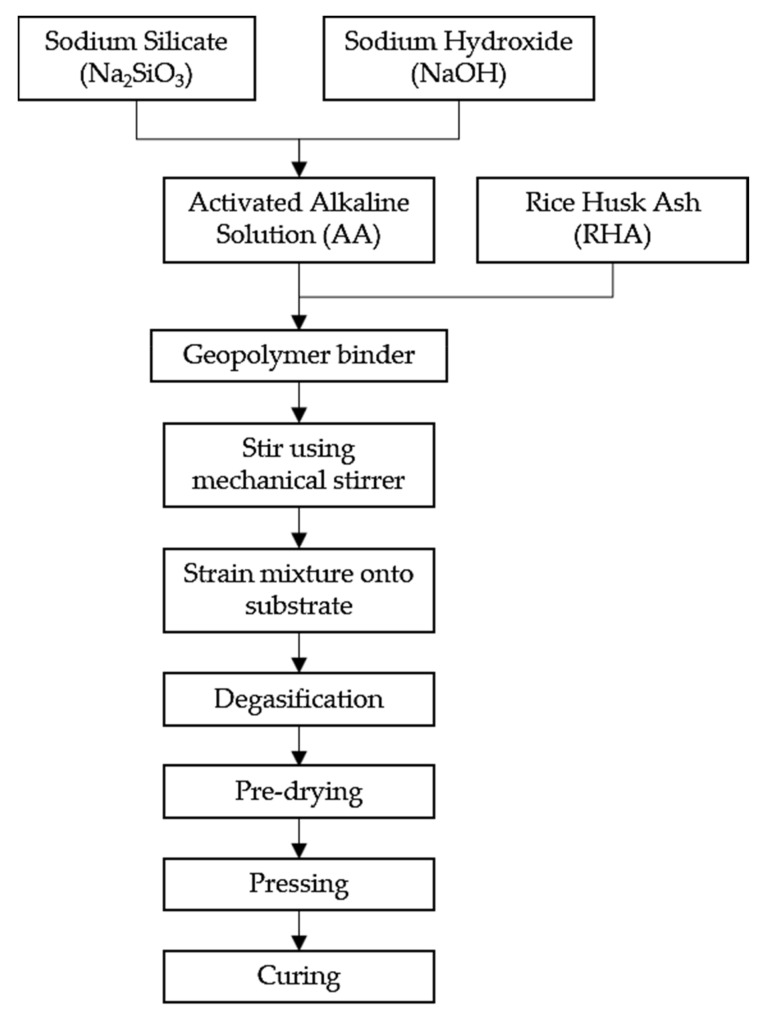
Flowchart for the fabrication of RHA-based geopolymer coating.

**Figure 4:**
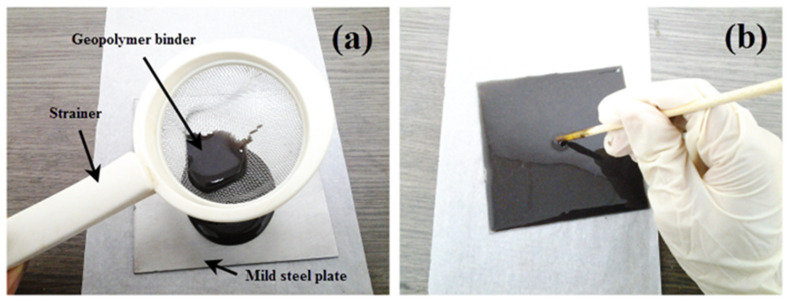
(**a**) Straining and (**b**) spreading the geopolymer binder.

Samples were prepared according to the flowchart, as shown in Figure 3. Na_2_SiO_3_ was added into NaOH solution at a ratio of 5.5 to form an activated alkali solution (AA) solution. The solution was then mixed with RHA at a designated ratio (Table 3) to obtain a dark gray slurry mixture. The mixture was then stirred with a mechanical stirrer (HS-300, WiseStir, Thessaloniki, Greece) at 150 rpm for 30 min, until homogenous. The mixture was strained through a small sieve directly onto the mild steel plate at 0.3 g/cm^2^ and was spread evenly, as shown in Figure 4.

The coated substrate was left for one minute for the coating binder to self-level. It was then placed in a vacuum oven (Model 53, Binder, Tuttlingen, Germany) for degasification to remove the remaining tiny bubbles. The coated substrate was pre-dried by placing it in an oven for approximately 40 min at 40 °C. The coated substrate was then removed from the oven and pressed by using a press machine (Gotech, Taichung City, Taiwan) to obtain the desired thickness. The thickness of the steel plate was 2 mm, which equaled the total thickness of the geopolymer binder coating (1 mm) and mild steel substrate (1 mm). The thickness was measured by using a digital Vernier caliper up to two decimal points accuracy. Then, the excess coating binder was trimmed, and the coated substrate was placed again in the oven to cure for 24 h at 50 °C. After 24 h, the coated substrate was left at room temperature for six days or more (depending on the experimental design) for complete curing. While geopolymer binder typically cures and hardens in 4 to 24 h, a longer curing duration was opted to ensure that the coatings were fully hardened and stabilized in ambient conditions.

### 2.4. Fire-Retardant Test

**Figure 5:**
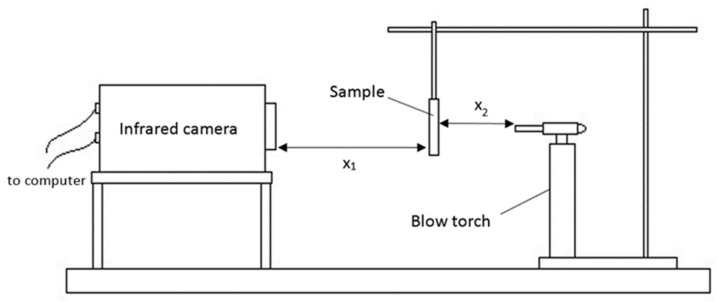
Setup for fire-retardant test.

The test was conducted by heating coated samples with direct blow torch flame following the UL-1709 standards [40]. The samples were heated directly, using a blow torch with flame temperatures around 900 °C. The specimens were kept 60 cm apart from the infrared camera (X_1_) and 7 cm from the blow torch (X_2_), as shown in Figure 5. The distance was determined based on previous literature and the safety of the infrared camera [41,42]. A shorter distance between infrared camera and specimens will result in camera overheat and damage. The distance over 7 cm will result in the blue flame not being in contact with the specimens, while the distance below 7 cm will result in the coating not having enough space to intumescent. At the beginning of every test, X_1_, X_2_, the ambient temperature and humidity were recorded on the computer. The recorded parameters were relevant since radiation was absorbed in the ambient space and transmittance was reduced with distance. Flame temperature versus time was taken as a result of fire protection in the experiment. A bare mild steel plate which acted as a control for the purpose of comparison was first exposed to direct flame for 10 min. A coated mild steel plate was exposed to direct flame for at least 20 min or until equilibrium temperature was reached.

### 2.5. Microstructure of Rice Husk Ash

A scanning electron microscope (SEM) (Hitachi, Tokyo, Japan) was used to analyze the difference in microstructure of samples before and after testing. SEM was conducted by using Hitachi S-3400N variable SEM. A total of five samples were taken, with two samples before the fire-retardant test, two samples after the test and one intumescent coating sample. The samples were first mounted with a conductive adhesive and sputter-coated with gold-palladium powder. The stub with sample specimens was inserted into the sample chamber of the SEM for viewing. Micrographs of sample surface were taken at magnifications of 100×, 200× and 1000×. Samples were also analyzed, using EDX, which is attached in the SEM, to determine elements in the specimens, including oxygen, silica, sodium, iron, carbon and calcium.

### 2.6. Thermogravimetry Analysis (TGA)

TGA is a method for measuring the weight loss of a material due to either increase in temperature or time. TGA was carried out by using SDTA 850 Mettler Toledo micro and ultra-micro balances in an atmosphere of flowing nitrogen gas in alumina crucible, at a heating rate of 10 °C/min over a temperature range from 50 to 1000 °C. Specimens, in the form of powder (RHA) and fine solid (geopolymer binder), weighing approximately 10 mg, were placed in crucibles.

### 2.7. X-ray Diffraction (XRD)

XRD was used to study the crystal structures of a material. Geopolymer residue samples and RHA were packed in circular cavity holders, and the phase composition of the samples was determined by using an Ital Structure APD 2000 diffractometer. Using a copper cathode in the 2θ angle range from 5° to 80°, the machine was operated with back monochrome running at 40 kW and 30 mA. The peak search for minerals in the diffraction data was conducted by using the PANalytical X’Pert HighScore software (version 1.0d). The software compared the d-spacing and intensity of each mineral with those of reference mineral standards.

### 2.8. Differential Scanning Calorimetric (DSC)

DSC measurements of geopolymer and RHA were conducted by using a Mettler Toledo DSC 823 with aluminum crucibles containing 5 to 10 mg of samples for the purpose of studying thermal transition following changes in a material, such as polymer, when it heated. The test was made under a dynamic nitrogen atmosphere at 60 mL/min and a heating rate of 5 °C/min in the temperature range of −30 to 160 °C. The DSC cell was calibrated with indium since it could identify heat of fusion and melting point efficiently. An empty pan sealed with a cover pan was used as a reference pan.

### 2.9. Coefficient of Thermal Expansion (CTE)

A dilatometer was used to monitor the CTE of the geopolymer sample expands or shrinks on heating. The test was conducted by using a DI-24 ADAMEL LHOMARGY dilatometer. Before testing, the samples were cast into a 1 mL syringe and cut to length to achieve a cylinder of 7.5 mm in diameter and 5 mm in length. The geopolymer samples were then positioned between two spacers, each measuring 5.0 mm in diameter and 0.7 mm thick. The alumina pushrod (piston) pushed the sample to position it next to the stationary alumina key. Once prepared, the sample holder was inserted into the interior of the combustion tube in the furnace and sealed by a high vacuum fitting. Measurement was conducted according to ASTM E831 standards between the temperature of 0 °C and 850 °C with a heating rate of 5 °C per minute.

## 3. Results and Discussion

The complete design matrix and response values of temperature at equilibrium (TAE) and time taken to reach 300 °C (TT300) are given in Table 5. Data were analyzed by using MINITAB. TAE depicts the fire-retardant properties of the geopolymer coating, where the temperature reached the maximum and was henceforth held constant. Low TAE indicates that the sample possesses good fire-retardant properties and vice versa. TT300 represents the time taken for the temperature to reach 300 °C. The longer the time taken the better is the good fire-retardant properties and vice versa.

### 3.1. Statistical Analysis of Temperature at Equilibrium and Time Taken to Reach 300 °C

A linear regression model was fitted to the experimental data, using the least-square technique. Several main parameters were considered in evaluating the statistical results, namely the coefficients of regression, the standardized error of coefficient, and the *p*-value of the effects of factors and its interaction for both responses, which are TAE and TT300. The results in Table 6 indicate that all factors and interaction effects are highly significant (*p* < 0.000) except for V_2_*V_2_ with *p* < 0.005. Values for R^2^ = 0.9522 and R^2^ (adjusted) = 0.9298 are considered high, indicating that 95.22% of the sample variation in the response was attributed to these factors.

For TAE, the *p*-values of the factors and their interactions were also considered significant when they was below the confidence level, which was also set at 95% (*p* of 0.050). The results shown in Table 7 indicate that all factors and interaction effects were significant. The *p* of all factors and interactions is highly significant (*p* < 0.000), except for V_2_ with *p* < 0.012. Values for R^2^ = 0.9754 and R^2^ (adjusted) = 0.9659 are considered very high, indicating that 97.54% of the sample variation in the response was attributed to the independent variables.

Equations (2) and (3) represent the regression models for the TAE and moisture absorption, respectively.

(2) $$\Upsilon_{TT300}=530.34-133.21\left(V_{1}\right)-81.10\left(V_{2}\right)-119.53\left(V_{1}{}^{2}\right)-56.22\left(V_{2}{}^{2}\right)+153.38\left(V_{1}V_{2}\right)$$

(3) $$\Upsilon_{TAE}=393.448+17.467\left(V_{1}\right)-6.708\left(V_{2}\right)+30.790\left(V_{1}{}^{2}\right)-36.075\left(V_{1}V_{2}\right)$$

where Υ_TT300_ and Υ_TAE_ represent the responses, which are TT300 and TAE, respectively. V_1_ and V_2_ are the decoded values of the RHA/AA ratio and NaOH concentration, respectively. The regression models can be used to calculate and analyze the effect of factors on the fire-resistance performance of RHA-based geopolymer coating.

### 3.2. Effect of Factors on Temperature at Equilibrium (TAE) and Time Taken to Reach 300 °C

**Figure 6:**
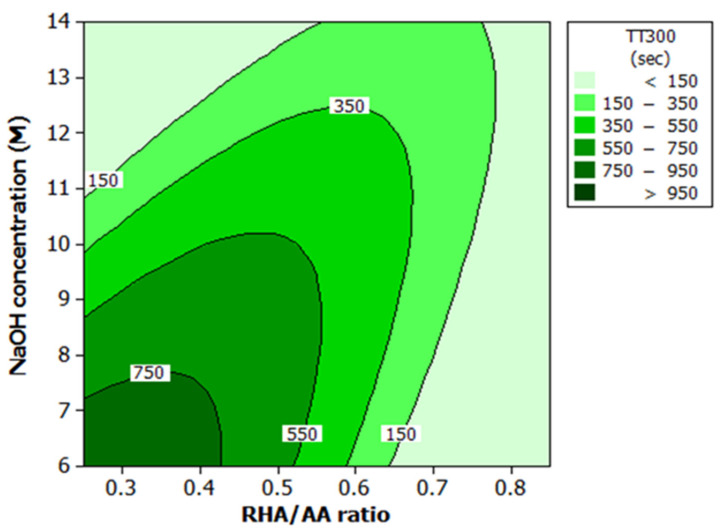
Contour plot for the effect of RHA/AA ratio and NaOH concentration on the TT300 in fire-retardant test.

**Figure 7:**
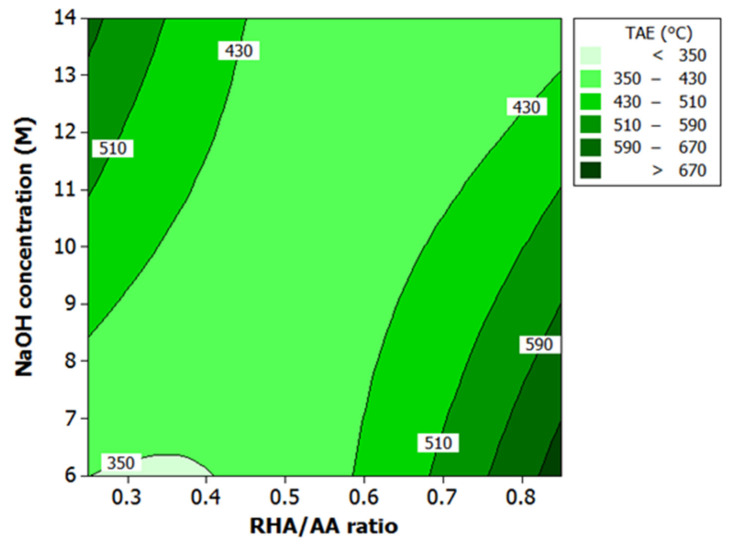
Contour plot for the effect of RHA/AA ratio and NaOH concentration on the TAE in fire-retardant test.

ANOVA and the regression models were used to analyze the effect of various factors on fire-resistance properties. Contour plots were used for better illustration. Figure 6 and Figure 7 illustrate the effect of the RHA/AA ratio (V_1_) and NaOH concentration (V_2_) on the responses. Both figures show that lower V_1_ and V_2_ resulted in a longer TT300 above 750 s and lower TAE at below 350 °C. According to Kuenzel et al. [43], a decrease in Na content decreases drying shrinkage sensitivity. In addition, a lower V_2_ contributes to overall low pH in the liquid phase due to the lower content of anionic form compared to molecular form. The coagulated structure is easier to form, since the liquid phase contains more stable molecular forms known as oligomers.

In addition, the rate of reaction is influenced by the mobility of the ions. As V_2_ decreases, the ion species concentration also decreases, allowing the dissolved ions to move freely. As a result, the units responsible for forming a covalently bonded network in the geopolymerization process meet faster, thus increasing the formation rate of the coagulated structures. This effect explains the higher rate of the geopolymerization process at a lower V_2_, as previously reported [44]. Zhang et al. [45] noted that the higher rate of geopolymerization is the reason for good thermal and mechanical performance. It is observed that the content of silica is inversely proportional to the V_1_. Lower V_1_ indicates higher Si/Al ratio. Figure 6 and Figure 7 show that a lower V_1_ contributes to better fire-resistance performance.

These results are associated with water and silica content in the geopolymer coating and are in agreement findings reported earlier [3,21]. According to Temuujin et al. [3], higher viscosity as indicated by low W/S ratio of FA-based geopolymer coating exhibited better thermal properties. Rickard et al. [21] found that geopolymer with higher silica content (higher Si/Al ratio) showed excellent thermal properties. This effect will be further explained in the following subsection.

### 3.3. Effect of Si/Al Ratio and W/S Ratio on the Responses

**Figure 8:**
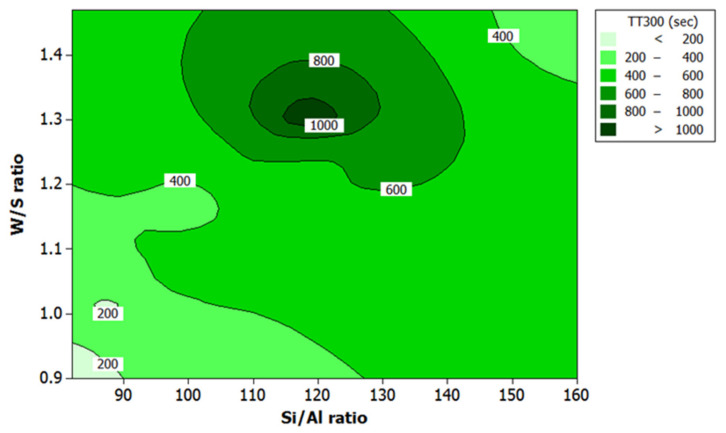
Contour plot for the effect of W/S ratio and Si/Al ratio on the TT300 in fire-retardant test.

**Figure 9:**
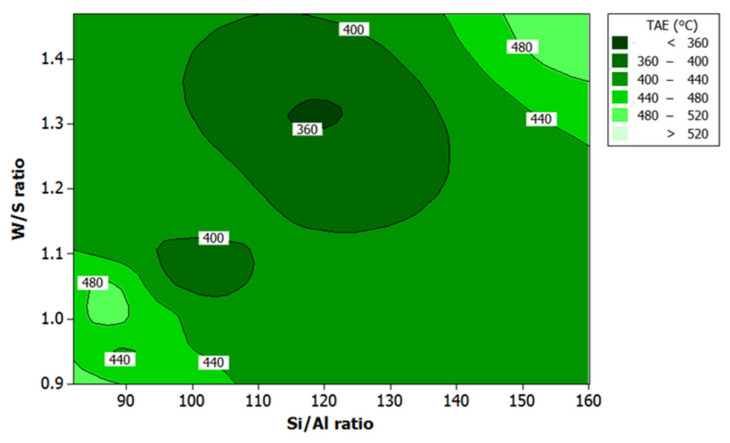
Contour plot for the effect of W/S ratio and Si/Al ratio on the TAE in fire-retardant test.

Figure 8 and Figure 9 show the contour plots of data on responses for all samples studied. These plots indicate a maximum fire-resistance performance in the region between 115 and 125 for Si/Al ratio and between 1.30 and 1.35 for W/S ratio for both responses. The results are in agreement with those reported earlier by Temuujin et al. [17] and Khan et al. [46].

The studies established that coating with good adhesion bonding resulted in good fire-resistance performance due to an increase in Si content. The high degree of dissolved Si thus created rougher surface [46]. Moreover, since the rougher surface is associated with good adhesion bonding, it can be concluded that geopolymer with a higher Si/Al ratio, such as sample S7 with a Si/Al ratio of 118.59, can produce better fire-resistance performance. In particular, the composition of the solution, which gave the maximum fire-resistance performance, has a microstructure that appeared glassy and not ‘smooth’ under scanning electron microscopy. In contrast, samples with a lower Si/Al ratio, such as sample S5 with Si/Al ratio of 88.95, have products with distinct cracks, although the surface appeared to be “smooth”, as shown in Figure 15.

The W/S ratio (between 1.30 and 1.35) produced maximum fire resistance. It meant that, when the W/S ratio was higher than 1.35, the geopolymer binder was more workable. However, more water had to be removed before the critical minimum water content was reached due to higher initial water contents, leading to coating delamination. The finding concurs with that reported earlier by Temuujin et al. [17].

A lower W/S ratio conversely will lead to low workability resulting in the improper mixing of the components and a less homogenous mixture. In addition, the high capillary pressure that develops between wet and dry areas of the micropore network leads to crack propagation in the microstructure due to the high capillary pressure [43]. According to Bhowmick and Ghosh [47], coating with high water content had low adhesion strength, relative to the steel substrate and higher expansion rate. Coating with good adhesion bonding can effectively protect the substrate without experiencing major early cracks during the curing process.

### 3.4. Optimization of the Responses

**Figure 10:**
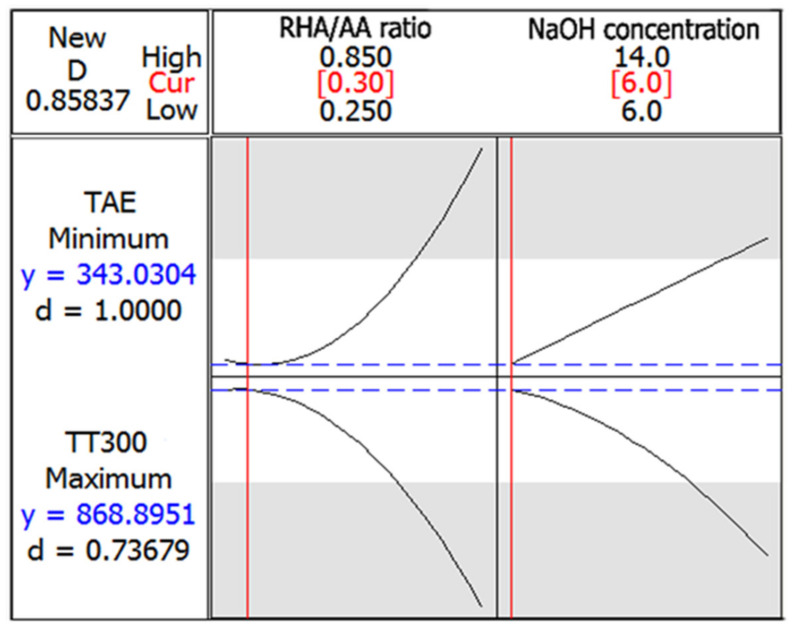
Optimization plot in fire-retardant test.

Figure 10 shows the optimization plot and the effect of different combinations of factor settings on the response. Since the objective was to minimize the temperature at equilibrium (TAE), the upper maximum acceptable value was set at 520 °C. The target value, which is the goal to achieve (in this case, the lowest temperature at equilibrium), was set at 345 °C. The maximum value was close to the highest value of TAE, and the target value was close to the lowest value of TAE.

For the time taken to reach 300 °C (TT300), the lower values of the minimum acceptable value were set at 110 s, since the objective was to maximize the TT300. The target value, which is the longest time taken to reach 300 °C, was set at 1140 s. The optimum TAE and TT300 values of 343 °C and 868 s, respectively, can be achieved with the combination of RHA/AA ratio (V_1_) = 0.30 and NaOH concentration (V_2_) = 6 M. The desirability of optimization was calculated as 0.85837, indicating that all parameters were within the target to obtain the maximum fire-resistance properties.

### 3.5. Experimental Validation

From Table 8, it was found that the average error for the TAE and TT300 was well below 15.00% at only 3.60% and 6.18%, respectively. It was concluded that the developed regression model that was established by using this method could optimize value for the responses accurately.

**Figure 11:**
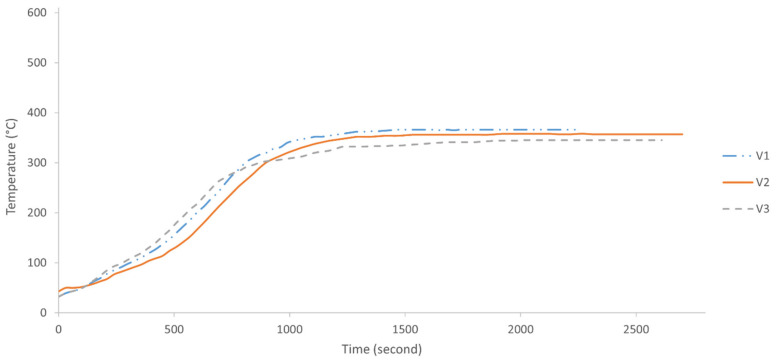
Graph temperature versus time of validated samples in fire-retardant test.

**Figure 12:**
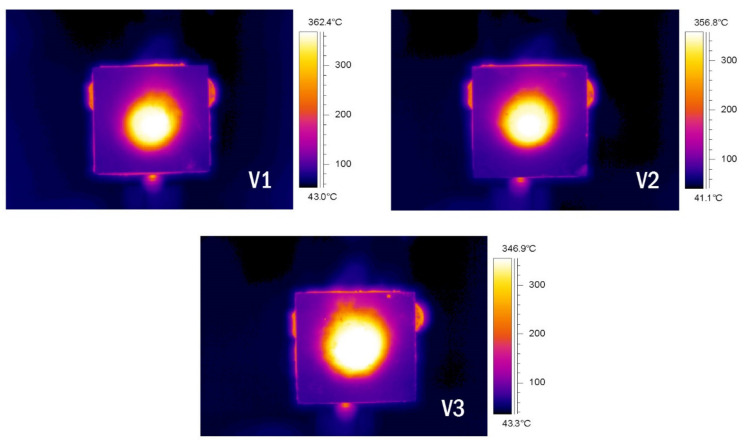
Thermal images of validated samples in fire-retardant test.

Figure 11 and Figure 12 illustrate the fire-retardant graph and thermal images of the validated samples, respectively. The graph reached equilibrium after approximately 20 min.

The thermal images show white regions, which represent a very high average temperature of 355.4 °C. These images confirmed that the heat that penetrated the outer coating and into the substrate was only confined to the central area. The coating appeared sufficiently efficient in controlling the heat from spreading to other parts of the substrate.

### 3.6. Material Characterization and Microstructural Analysis

Two samples, namely sample S7, which produced good fire-resistance performance, and S5, which had poor fire-resistance performance, were selected for further characterization and microstructural analysis

#### 3.6.1. X-ray Diffraction (XRD) Characterization

**Figure 13:**
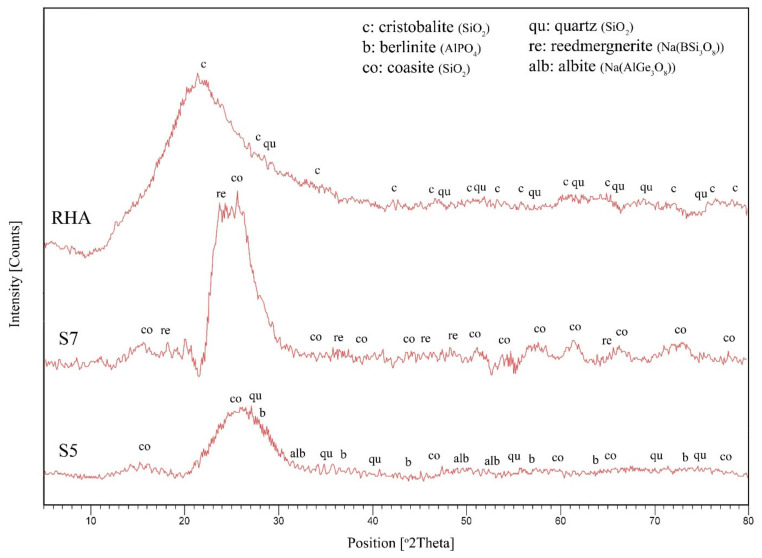
X-ray powder diffraction patterns of RHA, sample S7 and sample S5 before fire-retardant test.

The XRD patterns of RHA and cured geopolymer samples, which are samples S7 and S5 before the fire-retardant test, are shown in Figure 13. The RHA samples were from muffle furnace, from ash produced below 700 °C for 24 h burn. As evident from the diffractograms, the XRD patterns of the RHA show an amorphous halo.

Amorphous silica, a major constituent of the RHA, generates a broad XRD peak centered on a 2θ angle of 22°, which can be attributed to the presence of disordered cristobalite (SiO_2_) as similarly reported by Liou [48]. The burning of raw rice husks causes the decomposition of organic material and the breaking up of silicon bonds and the organic material. The Si-O groups become attached to produce a low form of cristobalite identified previously by Krishnarao and Godkhindi [49]. A very weak peak from quartz (SiO_2_), a crystalline phase of silica, is also found at this position. However, the height (intensity) is masked by the broad hump from the amorphous silica.

The results showed that silica in the RHA is mainly present as an amorphous phase with cristobalite and trace crystalline quartz. It is in agreement with the general literature [35,50]. According to Shinohara and Kohyama [35], tridymite and cristobalite are usually crystallized at temperatures ranging from 867 to 1470 °C and from 1470 to 1727 °C, respectively. However, due to the impure form of silica found in rice husk, the transition from amorphous to crystalline cristobalite and tridymite forms occurs at a much lower temperature [51]. The presence of impurities significantly reduced the transition temperature. When the starting materials are activated with an alkaline solution, the center of the original broad peak of RHA shifted from 22° to 26° 2θ, which is characteristic of geopolymer systems.

Delair et al. [52] found that this shift indicated changes in the local bonding environment during the geopolymerization process. The shift also indicated large-scale alteration and reconstruction of silica–alumina order in the paste, as strongly demonstrated in the geopolymerization, which occurred due to the amorphous nature of the samples. Temuujin et al. [3] determined that the width of the XRD halo in the geopolymer-type sample material suggests a mixture of unreacted RHA and the material. The halo width can be determined by measuring the full width at half the maximum (FWHM). The XRD halo of sample S5 was found broader compared to that of S7. Coesite (SiO_2_) and reedmergnerite (Na(BSi_3_O_8_)) were found in sample S7, while quartz (SiO_2_), albeit (Na(AlGe_3_O_8_)), coesite (SiO_2_) and berlinite (AlPO_4_) were found in sample S5.

The amorphous nature of S7 and S5 were also reported by Fletcher et al. [53]. They found that samples with high-silica composition range, which the Si/Al ratio between 2 and 300, were characteristically amorphous. In addition, it was found that the high intensity of the peak is also an indication of the high degree of crystallinity [54]. Although there is no scientific proof on the relationship between crystallinity and silica content in the geopolymer matrix, Table 9 shows that higher Si/Al ratio of sample S7 compared to S5 resulted in a higher peak. These results indicated that the high intensity in the XRD patterns was associated with the presence of coesite and not that of cristobalite thus suggesting the large presence of silica associated with RHA.

**Figure 14:**
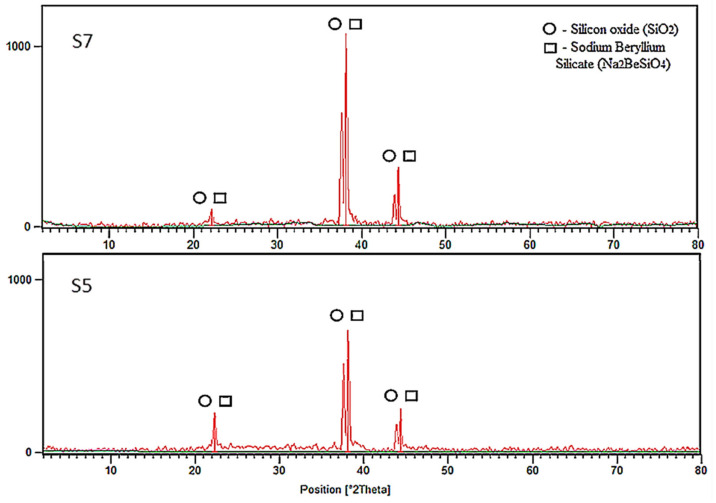
XRD patterns of samples S7 and S5 after fire-retardant test.

Figure 14 shows XRD patterns of the samples S7 and S5 after fire-retardant test. It is clear that, after exposure to a high flame temperature, crystallization occurred, and the samples changed from amorphous into crystalline phase. The presence of silicon oxide (SiO_2_) and sodium beryllium silicate was detected in both samples. The position of the broad peak shifted to the right, indicating that the alteration and reconstruction of silica–alumina order in the paste were extensive.

The pattern for S5 showed the presence of sodium and silica but in lower intensity than those in S7. Lower peak intensity was associated with a lower degree of crystallinity. Quartz (SiO_2_) and coasite (SiO_2_) detected in the geopolymer sample before the fire-retardant test has similar chemical compositions to SiO_2_. However, they have a different localized chemical environment which was influenced by pH, water content, Na concentration and Si species. The end product was expected to be different.

#### 3.6.2. Surface Morphology

**Figure 15:**
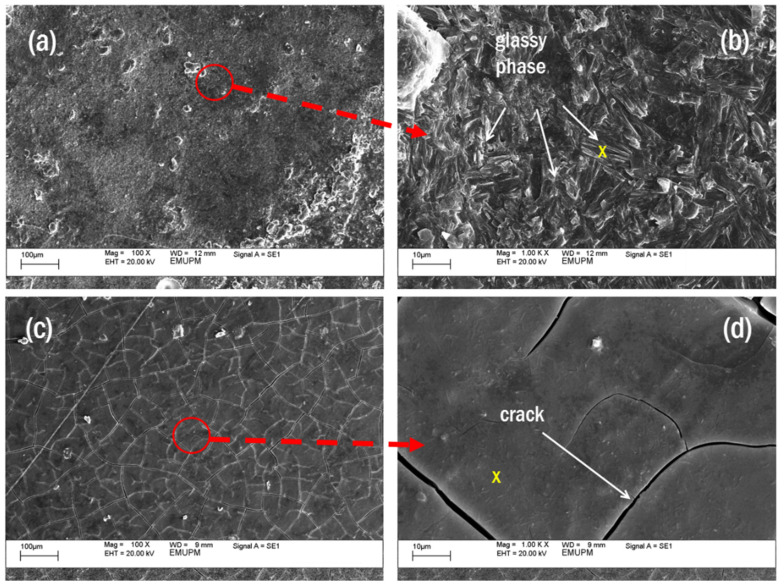
Thermal images of samples S7 (**a**,**b**) and S5 (**c**,**d**) in fire-retardant test.

Figure 15 shows scanning electron micrographs (SEM) of the microstructure of samples S7 and S5. Thermal images of validated samples in the fire-retardant test showed a glassy appearance that indicated a structure comprising a homogenous mixture of geopolymer elements, including Si and Na. It was evident in the EDX test result in Table 10. The yellow cross in Figure 15b,d indicated the area in which the EDX test was conducted for samples S7 and S5, respectively.

All results were presented in weight percent (wt.%). The results showed that the glassy phase in sample S7 was not due to an excess of sodium (Na) or silica (Si) but a good mix of the two components since the difference in wt.% between Na and Si was small, which was only 6.76%. This glassy phase was, thus, the result of a high degree of crystallinity, as indicated by the high peak intensity shown in Figure 14. Other elements found in sample S7 included oxygen and carbon.

A greater amount of unreacted particles was found in sample S5 as compared to S7, as denoted by a broader peak in the XRD graph shown in Figure 13. Compared to S7, it is also clear that the difference in wt.% between Si and Na content in sample S5 was higher, which is 23.58%, indicating that the RHA particles may not have fully dissolved. As studied by Temuujin et al. [17], the difference in microstructure greatly influences the physical properties, such as adhesion to the steel substrate.

**Figure 16:**
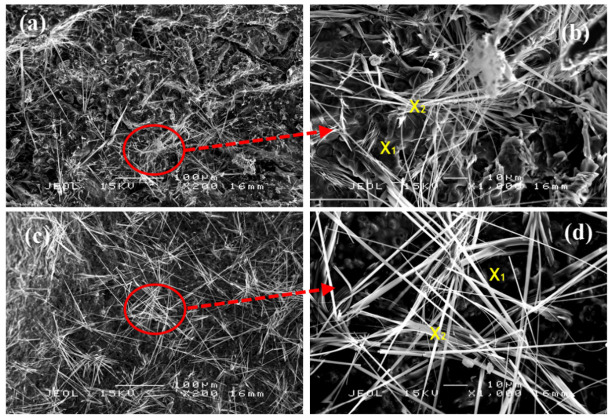
SEM micrographs of samples S7 (**a**,**b**) and S5 (**c**,**d**) and their magnification after the fire-retardant test.

Figure 16 and Table 11 show the SEM micrographs and EDX results, respectively, following the fire-retardant test. Samples for these tests were taken from the upper surface of the coating, which was directly exposed to the fire. The major elements present in the geopolymer binder were carbon (C), oxygen (O), sodium (Na) and silica (Si). EDX results on fire-retardant test of both samples, as illustrated in Table 11, showed an increase in carbon content and a decrease in oxygen content. The change in carbon and oxygen content was due to water evaporation and carbon formation during the test. The surface comprised a dense geopolymer gel, which was covered with long thin needle-like rod-shaped structures, as shown in Figure 16. Similar structures were reported in previous studies [55,56]. The long rod-shaped structures were a particular form of sodium derived from Na_2_SiO_3_ or due to a side reaction of NaOH and RHA, as shown by X_2_ mark in Figure 16b,d.

The EDX results in Table 11 show that point X_1_ indicated very high silica (Si) compared to sodium (Na) content. Point X_2_ in both samples had considerable presence of Na but low Si content with a trace of oxygen and carbon. Since Na_2_SiO_3_ is widely used as an intumescent thermal barrier coating material, as reported by Bulewicz et al. [57], it is possible to assume that these long-rod structures contribute to the fire-resistance properties of the coating. However, further analysis has to be conducted on this structure.

According to Zhao et al. [58], Si content in RHA plays a significant role in the fire-resistance properties. During the fire test, Si content accumulates in the very dense geopolymer gel, resulting in a silica-ash layer that acts as a heat barrier. This layer is vital in restricting oxygen access to the inner part of the coating, thus slowing down the gasification process. The sufficient loading, uniform dispersion and integrity of the silica-ash layer influence the effectiveness of the fire-resistance properties. As shown in Table 11, the content of Si at point X_1_ in the geopolymer gel of sample S7 was greater compared to that of S5. The RHA loading in the geopolymer gel was used to determine the thickness of the silica-ash layer. The effectiveness of the heat barrier increased proportionally with the thickness of this layer, as previously observed by Hshieh [59].

**Figure 17:**
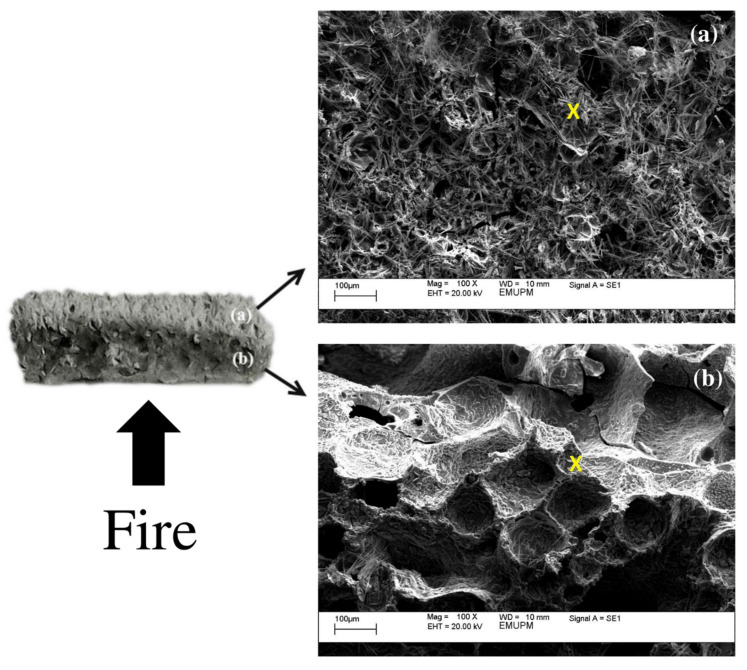
Color difference of (**a**) expended char and (**b**) residue across the thickness of the intumescent geopolymer coating after the fire test (sample S7).

In order to elucidate the fire-resistance properties of char in the intumescent coating, the residual chars burnt at around 850 °C were examined under SEM and EDX. Figure 17 shows two different types of structures across the thickness of the intumesced coating.

Part (a) of S7 was where the expanded char was immediately adjacent to the substrate and known as carbonaceous char. Part (b) of the sample, known as the residue, was nearer to the fire. Residual char refers to oxidized char, while carbonaceous char refers to char where carbon has not been oxidized.

According to Hirschler [60], besides the presence of residual and carbonaceous char, there was another layer called inorganic residue. The substance may form glassy layers which could be impenetrable to heat, thus protecting underlying layers from further thermal breakdown. The inorganic residue can exist alone or in combination with the carbonaceous char. Only with the presence of the inorganic residue, the carbonaceous chars are able to undergo oxidation under increasing temperature. It is, however, difficult to determine in Figure 17 whether part (a) is carbonaceous char (indicated by the presence of tiny pores) or a combination with inorganic residue (due to the development of long-rod structures that are crystal-like). The inorganic residue can also be the glassy layer created at the very top layer of the coating, as shown in Figure 20b.

Part (a) comprised tiny pores and long-rod structures believed to be a sodium–silica mixture. In addition to Na and Si, a small amount of carbon and calcium could be detected in the structure, as shown in Table 12. In part (b), the pores were around ten times larger than in part (a) and contained very low sodium and silica content. The carbon content was oxidized, and the released gasses included nitrogen and carbon dioxide. The large pores were probably developed under pressure existing inside the pores following water evaporation. The large pores may act as an effective first protection layer.

According to Gu et al. [61], the rate of protective char formation was greatly influenced by factors such as fire temperature and viscosity of the melting coating. Higher viscosity resulted in good char formation since it was difficult for gasses diffused during intumescence to escape from the char layer and nourish the flame. The compact spongy structures of part (a), laden with irregular mini-pores, function as an effective protective layer limiting heat transfer to the next layer, thus conserving the underlying material and consequently protecting the substrate.

#### 3.6.3. Thermogravimetric Analysis (TGA)

**Figure 18:**
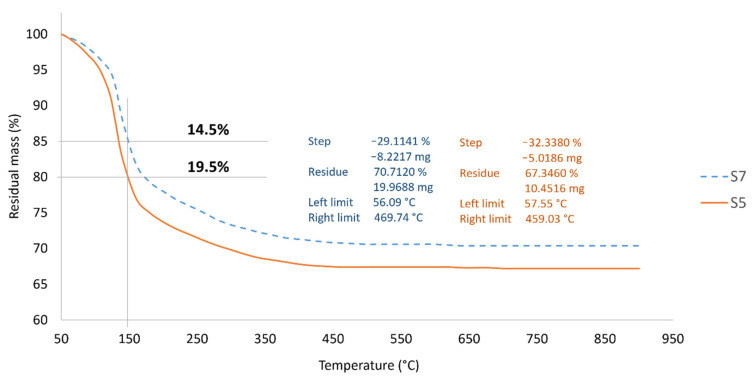
TGA of samples S7 and S5 in fire-retardant test.

Figure 18 shows TGA results obtained for samples S7 and S5 when exposed to nitrogen. Weight loss began at around 50 °C for both samples and a total of approximately 5.0% of absorbed water dehydrated at 120 °C. The onset of rapid weight loss occurred at about 120 °C, associated with the removal of free water, bonded water with hydrogen bond [62] or water bonded to silicate molecules [63].

According to Hollingbery and Hull [64], water loss through endothermic dehydration will leave a thermally stable residue. Since sample S5 has a higher dehydration than sample S7 with a 5.0% difference at 150 °C, the former reached thermal stability faster and at a lower temperature at 459 °C compared to sample S7 at 469 °C. The overall weight loss was 32.3% and 29.1% for samples S5 and S7, respectively. The results are quite similar to those recorded by Ferone et al. [65] for the TGA curve, using metakaolin-based geopolymer cured at room temperature. It is worth pointing out that metakaolin-based and RHA-based geopolymer coating may have similarities in terms of thermal degradation and stability.

According to Lyon et al. [66], the dehydration reaction results in loss of mass at temperature beyond 55 °C. This reaction equation also explains the intumescent process of the geopolymer coating. During the dehydration process, steam was produced at many times its liquid volume, and this created pressure. Duquesne et al. [67] reported that expansion or swelling of the intumescent coating is caused by slow diffusion of gas in the structure. When the intumescent coating expands, it provides “char” or insulating foam-like coating, which protects the substrate. In addition, the viscosity of the material must not be too high, which may cause the layer to break, or too low, which may stimulate gas evolution to feed the flame. The clarification is consistent with the results in Figure 8 and Figure 9 where the W/S ratio between 1.30 and 1.35 gave better results.

#### 3.6.4. Fire-Resistance Performance of Geopolymer Binder Coating

**Figure 19:**
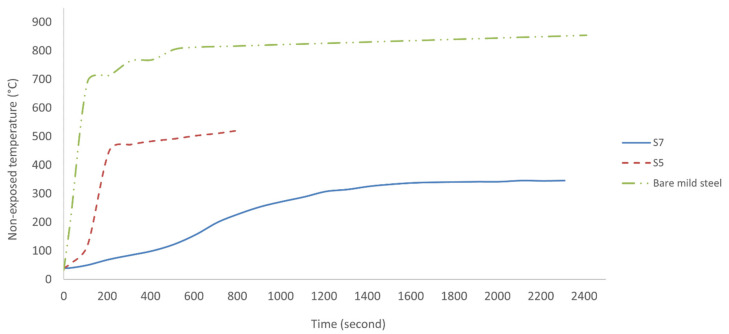
Result of fire-retardant test.

Figure 19 shows the fire-retardant test curves for bare mild steel, with sample S5 being poor in fire-resistance properties, and S7 the reverse. It should be considered that 500 °C is the temperature at which mild steel loses its strength, uncoated mild steel failed rapidly in only 28 s, while sample S5 failed after about 510 s when exposed to direct fire. The non-exposed surface temperature of mild steel plate in sample S7, which has the best coating composition, reached 300 °C after only 1113 s. In addition, the equilibrium temperature is approximately 347 °C, which is considerably lower than the failure temperature for mild steel after approximately 1500 s.

The rate of temperature increase at geopolymer–mild steel interface in sample S7 between the span of 60 to 540 s was only 11.4 °C/min, whereas between 540 and 690 s was 21.4 °C/min. The strong endothermic character of the material began to materialize between 540 and 690 s. After the fire test, the coating on sample S7 adhered strongly to the substrate, while in sample S5, it partially disintegrated. It was attributed to the intense creeping phenomena due to partial smelting of the material identified by Sakkas et al. [68]. According to Leiva et al. [69], the longer TT300 for sample S7 may be due to higher water content in the geopolymer paste. Sample S7 had water to solid (W/S) ratio of 1.30 compared to S5, with only 1.02. Due to the longer time it took for water to evaporate, S7 showed better thermal performance compared to S5.

**Figure 20:**
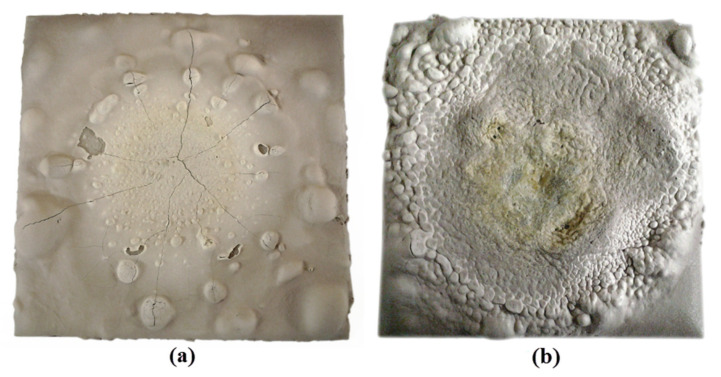
Mild steel plate coated with geopolymer binder for samples (**a**) S5 and (**b**) S7 after fire-retardant test.

Figure 20 shows mild steel plates coated with geopolymer following fire-retardant test. The crystal-like surface structure seen on sample S7 was most likely a silica-ash layer. Both samples underwent a development of the intumescent process, which can be divided into two steps based on Figure 19. The first step is the development of the intumescent process where the temperature sharply increased up to around 450 °C within 3 min and 320 °C within 25 min for samples S5 and S7, respectively. The second step is the intumescent shield plays its protective role where the temperature reaches an equilibrium state.

The development of the intumescent process was evident during the fire-retardant test. Once the coating surface was exposed to fire, it started to melt and became a highly viscous liquid. Chemical reactions took place, leading to bubble formation, producing swelling and a porous intumescent char layer. Cracks can be seen on the coating surface in sample S5, which probably led to poor fire-resistance performance. The sample exhibited very high thermal expansion, as seen in Figure 22, causing extreme mismatch, which loosened the interface bonding between coating and substrate. Cracks appeared after a slight collapse of the foam under gravity. The intumescent process observed was similar to that reported by Provis et al. [70].

In comparison, sample S7 was subjected to low thermal expansion and minimal mismatch with the substrate. Although there was slight shrinkage at the end of the test, the sample showed high structural integrity with no evidence of crack formation. The geopolymer coating was sufficiently flexible to cater to the minimal mismatch between substrate and coating. All samples did not release any smoke or ignite during the fire-resistance test regardless of the fire-resistance performance. The results showed that the coating was suitable for any application related to fire retardant and has great potential to replace existing coating materials, which are not environmentally friendly, in the market.

#### 3.6.5. Differential Scanning Calorimetric (DSC)

**Figure 21:**
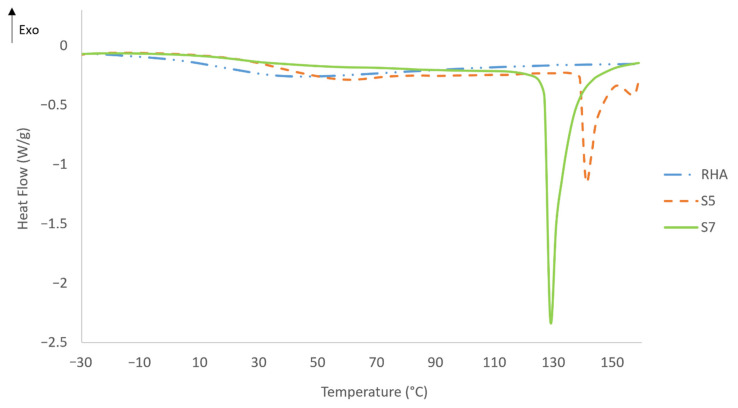
DSC result of RHA, sample S7 and sample S5 in fire-retardant test.

The DSC results for RHA samples S7 and S5 are plotted in Figure 21. The endothermic peak for both geopolymer samples corresponds with the portion of the DSC curve that dipped below the baseline for heat flow. The sharp dips shown for the two geopolymer samples indicate some degree of geopolymerization absent in the RHA. The DSC curve also serves as an indicator for water content and the energy absorbed by the samples.

Sample S7 has a larger area under the DSC curve, indicating that this sample has higher water content than S5. In the case of sample S5, it is observed that the dip in heat flow shifts slightly to a higher temperature. This may be due to the different forms of water present in the geopolymer samples, and the shorter time required for the sample to reach a specific temperature as discussed by Luna Galiano et al. [71]. This result is in agreement with the result of the fire-retardant test in Figure 19.

The energy absorbed correlates with the content and the chemical form in which free water, absorbed water, or crystallized water is present. According to Leiva et al. [69], higher water content in the geopolymer is associated with the energy absorbed by the sample. An earlier study by Vilches et al. [72] confirmed that the larger area under the DSC curve in sample S7 indicated an increase in the energy absorbed by the sample and a decrease in its thermal diffusivity. The smaller slope in S7 compared to that in S5 indicated a higher insulating capacity for the former sample material in Figure 19.

#### 3.6.6. Coefficient of Thermal Expansion (CTE)

**Figure 22:**
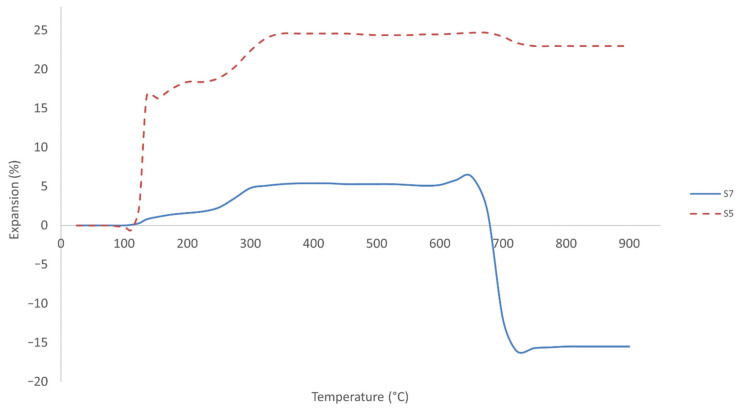
Thermal expansion characteristic of samples S7 and S5 in fire-retardant test.

To study the expansion behavior of the coated mild steel in samples S5 and S7, the CTE was measured, and the results are displayed in Figure 22.

Research on fly ash, metakaolin and kaolinite used as aluminosilicate sources has revealed thermal shrinkage but not the presence of the intumescent process [21,73]. Although the CTE test was conducted on only two samples in this study, all 26 samples exhibited thermal expansion, which is an intumescent-like expansion during the fire-retardant test. In order to produce an intumescent coating, sodium silicate, which is commonly used in the production of intumescent coating, must be combined with suitable aluminosilicate sources.

Sample S5 underwent a small degree of shrinkage of 0.5% at 112.4 °C due to dehydration of absorbed water, as shown in Figure 22. A previous study had noted that the presence of water was due either to absorbed water, which causes the network to swell, or to bound water in Si-OH groups [74]. Sample S7, however, showed no sign of shrinkage. Between 100 and 300 °C, coating expansion began to take place. Sample S5 exhibited a sharp expansion of nearly 22%. According to Provis et al. [70], this was probably due to relatively freely evaporation water loss and poorly formed geopolymer. As the dehydrating pore water escapes from the geopolymer gel due to the forces exerted by the highly-dense gel phase, fractures were created in the geopolymer gel. The coating began to expand, causing the cracks to propagate in the geopolymer gel, as discussed earlier by Rickard et al. [21]. Due to an increase in total pore volume, which generates very high pressure in the pores as the geopolymer gel shrunk, coating expansion occurred, creating cracks throughout the structure.

Between 100 and 300 °C, sample S7 exhibited nearly 5% expansion due to similar causes as for S5. Sample S7 had a higher proportion of geopolymer gel but a smaller amount of RHA particles due to a lower RHA/AA ratio of 0.40 compared to that of S5 with 0.70. The reacted and partially reacted particles were proportionally higher in S7, as shown in Figure 13. Since the bulk density was low, the porous structure was created by voids in between the geopolymer-coated RHA particles [75]. Consequently, less pore water was available for dehydration leading to fewer pores for expansion. The unreacted RHA particles were strongly bonded to the geopolymer gel, which was denser and less porous in both samples.

Between 300 and 600 °C, complete crystallization of the geopolymer surface and intumescent process occurred. Evaporated water was transported to the cooler area of the material, such as the inner part near the substrate. It condensed again, coalesced into a liquid film which induced an equilibrium in the temperature. Between 600 and 650 °C, both samples showed a slight expansion. It was assumed that water evaporation was completed at this stage, leaving a small portion of pores to expand slowly. However, the reasons behind the sudden expansion or shrinkage have not been studied. Above 650 °C, the pore structures began to collapse, causing the silica framework to soften, as discussed previously by Provis et al. [70].

## 4. Conclusions

A study investigating the fire-retardant and thermal properties, optimized composition and microstructural and element characteristics of rice-husk-ash-based geopolymer coating was successfully conducted. Response surface methodology (RSM) was successful in identifying the significant factors and in optimizing the responses. Results for the temperature at equilibrium (TAE) and time taken to reach 300 °C (TT300) showed that the RHA/AA ratio had the strongest effect on the responses. For the optimization process using RSM, the optimum value for both TAE and TT300 can be attained when the RHA/AA ratio is 0.30 and NaOH concentration is 6 M. The average error from the validation test for the TAE and TT300 was very low, at only 3.60% and 6.18%, respectively. SEM micrographs of good fire-resistance properties showed the following criteria: (i) it had a glassy appearance, and the surface coating changed into a dense geopolymer gel covered with thin needles when fired; (ii) it showed high insulating capacity; and (iii) it had a low thermal expansion, minimal mismatch with the substrate and the coating had no evidence of crack formation; and (iv) it had a low rate of dehydration. In general, the microstructure of GBC changed from amorphous to crystalline phase. When used as coating materials, RHA has proven to be the most effective alternative aluminosilicate source. The RHA-based geopolymer coating has the potential to improve fire safety in the construction of new buildings.

## Figures and Tables

**Table 1:** Total number of experimental runs for full factorial design and RSM based on 5-level factors.

Factors	Levels	Total Number of Experimental Runs	
		Full Factorial Design	RSM
4	5	625	31
5	5	3125	54
6	5	15,625	90
7	5	78,125	160

**Table 2:** Factors and levels.

Factor	Unit	Notation	Levels				
			−2	−1	0	1	2
RHA/AA ratio	-	V_1_	0.25	0.40	0.55	0.70	0.85
NaOH concentration	M	V_2_	6	8	10	12	14

**Table 3:** Design matrix.

Sample	Coded Factor		Uncoded Factor	
	V_1_	V_2_	V_1_	V_2_
S1	−1	1	0.40	12
S2	0	0	0.55	10
S3	−1	1	0.40	12
S4	0	0	0.55	10
S5	1	−1	0.70	8
S6	2	0	0.85	10
S7	−1	−1	0.40	8
S8	2	0	0.85	10
S9	1	−1	0.70	8
S10	0	2	0.55	14
S11	0	0	0.55	10
S12	0	0	0.55	10
S13	0	0	0.55	10
S14	0	0	0.55	10
S15	0	0	0.55	10
S16	−2	0	0.25	10
S17	−2	0	0.25	10
S18	1	1	0.70	12
S19	−1	−1	0.40	8
S20	0	−2	0.55	6
S21	0	0	0.55	10
S22	0	0	0.55	10
S23	1	1	0.70	12
S24	0	0	0.55	10
S25	0	−2	0.55	6
S26	0	2	0.55	14

**Table 4:** Physical properties of RHA after grinding.

Properties	RHA
Particles Size	<125 µm
Color	Light gray
Structure	Power form
Odor	Non

**Table 5:** Design matrix and response value for the temperature at equilibrium (TAE) and time taken to reach 300 °C (TT300).

Sample	RHA/AA Ratio (V_1_)	NaOH Concentration (V_2_)	RHA/AA Ratio (V_1_)	NaOH Concentration (V_2_)	TAE (°C)	TT300 (s)
S1	−1	1	0.40	12	394.0	520
S2	0	0	0.55	10	387.7	443
S3	−1	1	0.40	12	378.9	543
S4	0	0	0.55	10	398.8	400
S5	1	−1	0.70	8	527.5	153
S6	2	0	0.85	10	522.6	137
S7	−1	−1	0.40	8	348.3	1113
S8	2	0	0.85	10	510.8	117
S9	1	−1	0.70	8	519.7	237
S10	0	2	0.55	14	366.2	520
S11	0	0	0.55	10	394.0	333
S12	0	0	0.55	10	396.4	297
S13	0	0	0.55	10	396.3	447
S14	0	0	0.55	10	398.9	370
S15	0	0	0.55	10	405.0	420
S16	−2	0	0.25	10	512.5	303
S17	−2	0	0.25	10	516.7	300
S18	1	1	0.70	12	415.7	220
S19	−1	−1	0.40	8	349.2	1140
S20	0	−2	0.55	6	430.0	320
S21	0	0	0.55	10	394.0	340
S22	0	0	0.55	10	393.1	293
S23	1	1	0.70	12	418.3	207
S24	0	0	0.55	10	394.3	340
S25	0	−2	0.55	6	427.3	413
S26	0	2	0.55	14	358.0	617

**Table 6:** Estimated effects and coefficient for RHA/AA ratio and NaOH concentration on the TT300.

Term	Notation	Coefficient	Standard Error of Coefficient	*p*-Value
Constant		530.34	27.85	0.000
RHA/AA ratio	V_1_	−133.21	13.35	0.000
NaOH concentration	V_2_	−81.10	14.63	0.000
RHA/AA ratio*RHA/AA ratio	V_1_*V_1_	−119.53	17.35	0.000
NaOH concentration*NaOH concentration	V_2_*V_2_	−56.22	17.35	0.005
RHA/AA ratio*NaOH concentration	V_1_*V_2_	153.38	23.13	0.000

R^2^ = 0.9522; R^2^ (adj) = 0.9298.

**Table 7:** Estimated effects and coefficient for RHA/AA ratio and NaOH concentration on the TAE.

Term	Notation	Coefficient	Standard Error of Coefficient	*p*-Value
Constant		393.448	2.532	0.000
RHA/AA ratio	V_1_	17.467	2.179	0.000
NaOH concentration	V_2_	−6.708	2.387	0.012
RHA/AA ratio*RHA/AA ratio	V_1_*V_1_	30.790	1.780	0.000
RHA/AA ratio*NaOH concentration	V_1_*V_2_	−36.075	3.775	0.000

R^2^ = 0.9754; R^2^ (adj) = 0.9659.

**Table 8:** Experimental validation for the temperature at equilibrium and time taken to reach 300 °C.

Sample	TAE (°C)			TT300 (s)		
	Experimental Value	Predicted Value	Error (%)	Experimental Value	Predicted Value	Error (%)
SV1	362	343	5.54	822	869	5.41
SV2	357	343	4.08	928	869	6.79
SV3	347	343	1.17	924	869	6.33
	$\bar{x}$ Error		3.60	$\bar{x}$ Error		6.18

**Table 9:** Difference in Si/Al ratio, H_2_O and Na_2_O content of samples S7 and S5 after fire-retardant test.

Sample	Si/Al Ratio	H_2_O (mol)	Na_2_O (mol)
S7 (good)	118.59	0.423	0.043
S5 (poor)	88.95	0.348	0.035

**Table 10:** EDX result of samples S7 and S5 before fire-retardant test.

Element	S7	S5
	wt.%	wt.%
C	15.54	9.38
O	54.66	52.42
Na	11.52	7.31
Si	18.28	30.89

**Table 11:** EDX result of samples S7 and S5 measured at points X_1_ and X_2_ after fire-retardant test.

Element	S7 (X_1_)	S5 (X_1_)	S7 (X_2_)	S5 (X_2_)
	wt.%	wt.%	wt.%	wt.%
C	25.00	25.63	20.91	18.04
O	44.06	43.62	43.58	46.84
Na	0.28	2.87	28.98	26.47
Si	30.66	27.88	6.53	8.66

**Table 12:** EDX result of different area across the thickness of the intumescent geopolymer coating after fire test (sample S7).

Element	Part (a)	Part (b)
	wt.%	wt.%
C	12.62	-
O	54.46	65.92
Na	10.60	21.69
Si	22.04	12.40
Ca	0.28	-

## Data Availability

The data presented in this study are available on request from the corresponding author.
